# Supplementary material for: The Medicine Quality Monitoring Globe: A public health intelligence system for collecting and curating reports of substandard and falsified medical products from publicly available Internet news media
Source: PLOS Digit Health. 2026 Sep 15;5(9):e0001648. doi: 10.1371/journal.pdig.0001648 (PMC13577332; doi:10.1371/journal.pdig.0001648)
Supplement: Supplementary file 1 [file pdig.0001648.s001.docx]

Supplementary Material 1

**Queries of data source search engines**

As of January 2025, there are a total of 161 queries for English language, 201 for French language, 182 for Spanish language, 170 for Vietnamese language, and 297 for Mandarin used in Google News searches.

**Supplementary Material 1. List of search terms used**

| **Product-related search terms** | **Terms related to falsified or substandard quality** |
| --- | --- |
| **English**  antiprotozoal, antiparasitic, antihelminthic, anti-worm, antiseptic, antidiabetic, contraceptive, morning after pill, pregnancy test, condom, erectile dysfunction, uterotonic, myometrial, anti-androgen, anti-oestrogen, hormone, antiretroviral, anti-tuberculosis, vaccine, laxative, antacid, antispasmodic, cardiovascular, cardiac, diuretic, anti-arrhythmic, antihypertensive, antianginal, vasoconstrictor, anticoagulant, antiplatelet, antifibrinolytic, bronchodilator, corticoid, corticosteroid, antihistamine, mucolytic, cough suppressant, decongestant, sedative, anxiolytic, benzodiazepine, antipsychotic, antidepressant, anti-obesity, analgesic, antimigraine, antiepileptic, anticonvulsant, antiparkinsonian, antimuscarinic, antithyroid, chemotherapy, cytotoxic, immunosuppressants, antibodies, vitamin, anti-inflammatory, anesthetic, anaesthetic, medicine, drug, medication, pharmaceutical, injection, parenteral, tablet, capsule, pill, cream, gel, antibacterial, antibiotic, antimicrobial, antiviral, antifungal, abortive, myometrial relaxant, hypnotic, immunoglobulin, veterinary medicine, medical device, laboratory reagent, chemicals, antimalarial, lipid lowering, cholesterol lowering, painkiller, pain-reliever, blood thinner, glucose lowering, sleeping pill, sleeping tablet, jab, anticancer, antimigraine, tranquilizer, clotting agent, blood sugar lowering, electrolytes, blood pressure lowering, disinfectant, drip, fat level decreasing, cough reliever, gammaglobulin, anti-seizures, parkinson medicine, worming medicine, hormone replacement therapy, contrast dye, insecticide, pesticide, repellents, fly spray, alzheimer medicine, weight loss medicine, ophthalmic medicine, nutritional supplement, food supplement, anti-allergy, antipyretic, eye drops, ear drops, diagnostic, covid, cv-19, cv19, coronavirus, sars, mask, hand sanitizer, hand sanitizing, hand sanitiser, gloves, ventilator, testing kit, test kit, pcr test, facemask, facemasks, syringe, needle, vial, oxygenator, oxygen, pulse oximeter, cpap, continuous positive airway pressure, peep, positive end-expiratory pressure, bag-valve-mask, ambu bag, bag valve, self-inflating bag, nebulizer, nebuliser, nasal cannula, nasal catheter, oropharyngeal catheter, manual resuscitator, hepa filter, intubation kit, endotracheal tube, tracheal tube, tracheostomy tube  **Specific search query for stolen/diverted COVID-19 vaccines:**  (vaccine OR vaccines) AND (Covid-19 OR Covid19 OR CV19 CV-19 OR covid OR SARS-CoV-2 OR SARS OR Cov-2 OR coronavirus)  AND (theft OR thefts OR diversion OR diversions OR stolen OR robbery OR thievery OR diverted)  **Exclusion terms**  English: diluted share, diluted earnings, diluted loss per share, fake news, diluted eps, eps | **English**  Counterfeit, fake, Spurious, Substandard, Falsified, Degraded, Contaminated, Adulterated, "Drug quality", "Medicine quality", "Pharmaceutical quality", "Falsely labelled", Rogue, Bogus, Quack, Diluted, "Poor quality", "Low quality", tamper, fraud, fraudulent |
| **Spanish**  antiprotozoario, antiparasitario, antihelmíntico, antiséptico, antidiabético, contraceptivo, píldora del día después, test de embarazo, condón, disfunción eréctil, miometrial, antiandrógeno, antiestrógeno, hormona, antirretroviral, antituberculoso, vacuna, laxante, antiácido, antiespasmódico, cardiovascular, cardiaco, diurético, antiarrítmico, antihipertensivo, vasocontrictor, anticoagulante, antiagregante, broncodilatador, corticoide, corticosteroide, antihistamínico, mucolítico, antitusivo, descongestionante, sedante, ansiolítico, benzodiacepina, antipsicótico, antidepresivo, anti-obesidad, analgésico, antimigrañoso, antiepiléptico, anticonvulsivante, antiparkinsoniano, antitiroideo, quimioterapia, citotóxico, inmunosupresor, anticuerpos, vitamina, antiinflamatorio, anestésico, medicina, droga, medicación, farmacéutico, inyección, parenteral, pastilla, cápsula, píldora, crema, gel, antibacteriano, antibiótico, antimicrobiano, antiviral, antifúngico, abortivo, hipnótico, inmunoglobulina, medicina veterinaria, aparato médico, productos químicos, antipalúdico, analgésico, anticoagulante, hipoglicemiante, pastilla para dormir, vacuna, anticanceroso, tranquilizante, hipoglicemiante, electrolito, antihipertensivo, desinfectante, suero, hipolipemiante, antitusivo, gammaglobulina, antiepiléptico, terapia de reemplazo hormonal, intravenoso, insecticida, pesticida, repelente, antimosquito, suplemento nutricional, suplemento alimenticio, antialérgico, antipirético, gotas de ojos, gotas de oídos, diagnóstico, impotencia, antiandrogénico, antiestrogénico, antirretrovírico, sedativo, farmacéutica, ungüento, antimicótico, calmante, antineoplásico, gotero, adelgazante, suplemento alimentario, gotas oculares  **Specific search query for stolen/diverted COVID-19 vaccines:**  vacuna AND (COVID-19 OR COVID OR cv-19 OR cv19 OR cv OR coronavirus OR SARS-CoV-2 OR SRAS OR CoV‑2) AND (desviado OR desviada OR desvíos OR desviaciones OR robo OR hurto OR sustracción OR saqueo OR Robado OR hurtado OR sustraido OR saqueado OR robos OR hurtos OR sustracciones OR saqueos)  **Exclusion terms**  Spanish: noticias falsas, bulos | **Spanish**  Falso, Espurio, Substandard, Falsificado, Degradado, Contaminado, Adulterado, "Calidad de los medicamentos", "Calidad de las medicinas", Corrupto, Deshonesto, Diluído, "Mala calidad", "Baja calidad", Alterar, Corromper, Fraude, Fraudulento |
| **Chinese Google**  CoV‑2, PCR检测, SARS, SARS-CoV-2, 不经肠的, 丙种球蛋白, 丸剂, 乳液, 乳霜, 事后避孕丸, 事后避孕药, 保健品, 保险套, 假疫苗, 假药, 催眠, 催眠剂, 催眠术的, 免疫抑制剂, 免疫球蛋白, 免疫血球素, 兽医, 兽药, 农药, 冠状病毒病, 冠狀病毒病, 减充血剂, 减肥, 减肥药, 凝胶, 凝血剂, 利尿剂, 利尿的, 制药, 制酸剂, 勃起功能障碍, 勃起障碍, 化学物, 化学物品, 化学物质, 化学疗法, 化学药品, 化学药物治疗, 化工原料, 化疗, 化痰剂, 医学, 医疗器材, 医疗器械, 去充血剂, 发育不全的, 口咽导管, 口服避孕药, 口罩, 呼吸机, 呼气末正压, 咳嗽抑制剂, 喷蝇油, 喷雾器, 堕胎, 奶油, 奶油色, 妊娠测试, 妊娠试验, 子宫收缩剂, 子宫收缩药, 子宫肌层的, 子宫肌松药, 子宫肌的, 安全套, 安定剂, 安定药, 安眠药, 安眠药片, 实验室试剂, 实验试剂, 宫缩药, 对比染料, 帕金森医学, 帕金森药物, 心脏的, 心脏血管, 心血管, 性功能障碍, 手动复苏器, 手套, 打胎, 打针, 抑制精神的, 抑菌, 抗体, 抗偏头痛, 抗偏头痛剂, 抗偏头痛药, 抗凝剂, 抗凝血, 抗凝血剂, 抗原生动物, 抗原虫, 抗反转录病毒, 抗发炎, 抗寄生物的, 抗寄生物药, 抗寄生虫, 抗巴金森症药, 抗帕金森病, 抗微生物剂, 抗心律不齐药, 抗心律失常, 抗心律失常药, 抗心绞痛, 抗惊厥, 抗惊厥药物, 抗抑郁剂, 抗抑郁的, 抗抑郁药, 抗敏, 抗炎, 抗炎的, 抗焦虑药, 抗生素, 抗甲状腺, 抗甲状腺剂, 抗甲状腺药物, 抗甲腺, 抗疟疾, 抗疟的, 抗疟药, 抗疟药物, 抗病毒的, 抗痉挛, 抗痉挛药, 抗痨, 抗癌剂, 抗癌的, 抗癌药, 抗癫痫, 抗癫痫药物, 抗癫药, 抗真菌的, 抗精神病药, 抗糖尿病的, 抗糖尿病药, 抗纤溶, 抗纤维蛋白溶药, 抗纤维蛋白溶解的, 抗组织胺, 抗组织胺药, 抗组胺, 抗组胺剂, 抗细菌, 抗结核, 抗肥胖, 抗肥胖药物, 抗菌, 抗菌剂, 抗菌素, 抗蠕虫, 抗血小板, 抗过敏的, 抗逆转录病毒, 抗酸药, 抗雄激素, 抗雄激素物质, 抗雌激素, 抗雌激素物质, 抗高血压, 持续气道正压通气系统, 指尖脉搏血氧仪, 插管工具, 支气管扩张剂, 支气管扩张药, 新冠肺炎, 服药, 杀真菌的, 杀菌剂, 杀菌性, 杀虫剂, 杀虫药, 检测盒, 止咳药, 止疼药, 止痛剂, 止痛片, 止痛的, 止痛药, 毒品, 气囊阀, 气管切开术管, 气管导管, 氧合器, 氧气, 治疗偏头痛, 治疗偏头痛药物, 治疗心绞痛, 治糖尿病药, 注射, 注射剂, 注射器, 泻剂, 泻药, 洗手液, 流产的, 消毒剂, 消毒洗手液, 消毒的, 消炎药, 溶解黏液, 滴注器, 滴眼剂, 滴眼液, 滴耳剂, 滴耳液, 激素, 激素取代疗法, 激素替代治疗, 灭蝇喷射油, 点滴, 片剂, 瓶, 电解液, 电解物, 电解质, 疫苗, 皮质甾类, 皮质类固醇, 眼科医学, 眼药水, 祛痛, 祛痰药, 类皮质激素, 粘液溶解, 精神抑制药, 细胞干扰素, 细胞毒素的, 维他命, 维生素, 老年痴呆症医学, 耳液, 耳药水, 肌松药, 肠外, 肠胃外的, 胶, 胶体, 胶囊, 脂肪水平下降, 自充气袋, 苯二氮, 苯二氮卓, 苯二氮卓类药物, 药, 药丸, 药剂, 药品, 药品质量, 药片, 药物, 药物治疗, 药瓶, 药膏, 荷尔蒙, 营养品, 营养补剂, 蠕虫药, 血液稀释剂, 血管收缩剂, 血管收缩的, 血管收缩药物, 解热, 解热剂, 解热药, 解痉药, 解酸剂, 解酸药, 诊断, 轻泻剂, 退烧药, 退热, 通便, 避孕, 避孕剂, 避孕套, 避孕药, 针, 镇咳剂, 镇定剂, 镇痉剂, 镇痛剂, 镇痛药, 镇静剂, 镇静药, 防腐剂, 防酸剂, 阳痿, 阿尔兹海默医学, 阿尔兹海默药物, 降低胆固醇, 降压, 降压药物, 降糖, 降糖药物, 降胆固醇, 降脂, 降血压, 降血糖, 非典, 非肠道的, 预防针, 驱肠虫, 驱虫剂, 驱虫药, 验孕, 高效空气过滤器, 麻药, 麻醉, 麻醉剂, 麻醉药, 鼻套管, 鼻导管  **Specific search query for stolen/diverted COVID-19 vaccines:**  疫苗 AND (新冠 OR 新冠病毒 OR 新冠疫情 OR 新型冠状病毒肺炎 OR 武汉新型冠状病毒 OR 武汉肺炎 OR 新型冠状病毒 OR SARS-CoV-2 OR SARS OR 非典 OR CoV‑2) AND (转移 OR 贸易转移 OR 盗窃 OR 抢劫 OR 偷 OR 偷窃)  **Exclusion terms**  Mandarin (GoogleNews): 假新闻,股票,证券,股市,股份  Mandarin (Baidu) : 股票,证券,股市,股份  **Mandarin Chinese – Baidu**  药品质量, 假药, 假疫苗 | **Chinese Google**  假冒的, 假的, 假的, 伪造的, 欺骗性的, 劣质的, 伪造的, 降级的, 被污染的, 参假的, 药品质量, 标识假的, 捏造的, 稀释的, 品质低劣的, 低质量的, 伪劣品, 冒牌货, 欺骗的 |
| **French**  antiseptique, antiprotozoaire, antiparasitaire, anthelminthique, vermifuge, anti-diabétique, pillule du lendemain, test de grossesse, préservatif, dysfonction érectile, utérotonique, anti-androgène, hormone, antirétroviral, anti-tuberculeux, vaccin, laxatif, antiacide, anti-spasmodique, cardiovasculaire, cardiaque, diurétique, anti-hypertenseur, vasoconstricteur, anticoagulant, antiplaquettaire, antifibrinolytique, bronchodilatateur, corticoïde, corticostéroïde, anti-histaminique, mucolytique, antitussif, décongestionnant, sédatif, anxiolytique, antidépresseur, anti-obésité, analgésique, antimigraineux, antiépileptique, anticonvulsivant, antiparkinsonien, antithyroïdien, chimiothérapie, cytotoxique, immunodépresseur, anticorps, vitamine, anti-inflammatoire, anesthésiant, anesthésique, médicament, drogue, médication, thérapeutique, injection, parentéral, comprimé, gélule, pillule, crème, gel, antibactérien, antibiotique, antimicrobien, antiviral, antifongique, abortif, hypnotique, immunoglobuline, médicament vétérinaire, dispositif médical, produits chimiques, antipaludique, hypolipidémiant, hypocholestérolémiant, calmant, antidouleur, fluidifiant, hypoglycémiant, somnifère, piqûre, anticancéreux, tranquillisant, médicaments contre le diabète, électrolytes, médicaments contre la tension, désinfectant, gammaglobuline, produit de contraste, insecticide, pesticide, répulsif, anti-Alzheimer, médicament pour maigrir, complément alimentaire, anti-allergique, antipyrétique, collyre, gouttes auriculaires, diagnostique, antidiabétique, contraceptif, trouble de l'érection, antiandrogène, antituberculeux, antispasmodique, antiarrythmique, antihypertenseur, antiaggrégant, antihistaminique, antipsychotique, antiobésité, anticholinergique, antinéoplasique, immunosuppresseur, remède, perfusion, cachet, onguent, antipaludéen, aide-minceur, gouttes ophtalmiques, solution auriculaire, Impuissance, anticonvulsif, médicament contre l'obésité, antalgique, antitumoral, pastille, pommade, coupe-faim, gouttes pour les yeux, gouttes oculaires, spasmolytique, hypoglycémique, COVID, CV-19, CV19, coronavirus, SARS‑CoV‑2, CoV‑2, SRAS, masque, désinfectant pour les mains, désinfection des mains, gants, ventilateur, kit de test, trousses de dépistage, kit de dépistage, test PCR, seringue, aiguille, flacon, ampoule, fiole, oxygénateur, Oxygène, oxymètre de pouls, PEP, Nébuliseur, filtre HEPA, sonde trachéale, Canule de trachéotomie  **Specific search query for stolen/diverted COVID-19 vaccines:**  vaccin AND (COVID-19 OR COVID OR coronavirus OR SARS‑CoV‑2 OR SRAS OR CoV‑2) AND (détourné OR détournements OR brigandage OR Pillage OR cambriolage OR volé OR volée OR volés OR volées OR vols OR larcins OR vol OR larcin)  **Exclusion terms**  French: Fausses information | **French**  contrefaçon, contrefait, faux, sous-standard, degradé, adultéré, contaminé, "qualité du médicament", falsifié, fallacieux, "qualité pharmaceutique", fraude, frauduleux, "faussement étiqueté", "qualité inférieure", "mauvaise qualité", imitation |
| **Vietnamese**  chống động vật nguyên sinh, diệt ký sinh trùng, diệt giun sán, tẩy giun, sát khuẩn, trị đái tháo đường, Tránh thụ thai, morning after pill, Thử thai, Bao cao su, Rối loạn cương dương, Co bóp tử cung, kháng androgen, kháng estrogen, nội tiết tố, Kháng virus, Chống lao, vắc-xin, nhuận tràng, kháng axit, chống co thắt, tim, lợi tiểu, chống loạn nhịp tim, hạ huyết áp, Thuốc co mach, chống đông máu, kháng tiểu cầu, thuốc tiêu sợi huyết, thuốc giãn phế quản, thuốc corticoid, Kháng histamine, long đờm, giảm ho, thông mũi, an thần, giảm lo âu, thuốc benzodiazepin, chống loạn thần, chống trầm cảm, chống béo phì, thuốc giảm đau, chống đau nửa đầu, chống động kinh, chống co giật, thuốc điều trị Parkinson, kháng giáp, hóa trị, gây độc tế bào, ức chế miễn dịch, kháng thể, vitamin, chống viêm, gây mê, thuốc, dược phẩm, tiêm, viên, kem, gel, kháng khuẩn, kháng sinh, kháng vi sinh vật, kháng vi-rút, kháng nấm, phá thai, giãn tử cung, thuốc ngủ, globulin miễn dịch, thuốc thú y, thiết bị y tế, thuốc thử, hóa chất, thuốc điều trị sốt rét, hạ lipid máu, hạ cholesterol, thuốc giảm đau, làm loãng máu, hạ glucose, chống ung thư, tác nhân đông máu, hạ đường máu, hạ huyết áp, thuốc khử trùng, nhỏ giọt, giảm lượng mỡ, gamma globulin, liệu pháp thay thế hormone, thuốc nhuộm tương phản, thuốc trừ sâu, thuốc diệt cỏ, thuốc chống côn trùng, bình xịt côn trùng, Thuốc trị bệnh Alzheimer, thuốc giảm cân, thuốc nhãn khoa, thực phẩm chức năng, chống dị ứng, hạ sốt, thuốc nhỏ mắt, thuốc nhỏ tai, chẩn đoán, trị ký sinh trùng, chống giun, khử trùng, trị tiểu đường, Tránh thai, Cơ tử cung, trị lao, chống rối loạn nhịp tim, chống tăng huyết áp, chống kết tập tiểu cầu, chống đông máu, xịt mũi, gây ngủ, liệu pháp hóa học, thuốc ức chế miễn dịch, gây tê, chống vi khuẩn, kháng virus, hỏng thai, thuốc chống sốt rét, thuốc sát khuẩn, thuốc diệt côn trùng, chống đái tháo đường, ngừa thai, COVID, CV-19, CV19, vi rút corona, SARS-CoV-2, CoV-2, SARS, khẩu trang, nước rửa tay, khử trùng tay, Găng tay, Máy thở, kit xét nghiệm, Xét nghiệm PCR, xi lanh, bơm tiêm, ống, ống tiêm, Máy tạo oxy, ôxy, oxy, Máy đo nồng độ oxy, Máy thở áp lực dương liên tục, CPAP, Thở áp lực dương liên tục, PEEP, Áp lực dương tính cuối thì thở ra, Túi van mặt nạ, BVM, Túi ambu, Túi van mặt nạ, Máy khí dung, máy xông khí dung, ống thông mũi, ống thông mũi, ống thông hầu họng, máy hồi sức thủ công, Bộ lọc HEPA, bộ đặt nội khí quản, dụng cụ đặt nội khí quản, ống nội khí quản, ống khí quản, ống mở khí quản  **Specific search query for stolen/diverted COVID-19 vaccines:**  (vắc-xin OR vaccine) AND (COVID-19 OR vi rút corona OR SARS-CoV-2 OR SARS OR CoV-2) AND (chuyển hướng OR sự chuyển hướng OR ăn trộm OR trộm OR ăn cướp OR cướp OR ăn cắp OR trộm cắp)  **Exclusion terms**  Vietnamese: tin tức giả | **Vietnamese**  "giả mạo" , giả , "dưới tiêu chuẩn" , "không đạt tiêu chuẩn" , "kém chất lượng" , "chất lượng kém" , "nhiễm tạp" , "bị lẫn" , "pha trộn" , "chất lượng thấp" , "không đạt chất lượng" , "chất lượng thuốc" , "lừa đảo" , "không có thật" , "lừa gạt" , "pha loãng" , "gian lận" |
