# Supplementary material for: The Medicine Quality Monitoring Globe: A public health intelligence system for collecting and curating reports of substandard and falsified medical products from publicly available Internet news media
Source: PLOS Digit Health. 2026 Sep 15;5(9):e0001648. doi: 10.1371/journal.pdig.0001648 (PMC13577332; doi:10.1371/journal.pdig.0001648)
Supplement: Supplementary file 2 [file pdig.0001648.s002.docx]

Supplementary Materials 2

**COVID-19 ‘Shortcut queries’ in the MQM Globe and associated key terms**

Due to COVID-19 pandemic, the MQM Globe search box was adapted to enable quick access to reports of COVID medicines, Personal protective equipment (PPE), Sanitisers and disinfectant, COVID vaccines, Ventilation and Oxygenation, and COVID diagnostics.

Available COVID-19 shortcuts queries on the MQM Globe web app

| **Shortcut name** | **Returns** |
| --- | --- |
| COVID medicines | Medicines used to treat COVID-19 and experimental drugs in clinical trials. Includes biologics such as monoclonal antibodies. |
| Personal Protective Equipment | Gowns, gloves, facemasks, visors, etc. |
| Sanitisers and disinfectant | Wipes, disinfectant, sanitizer, etc. |
| Ventilation and oxygenation | Continuous Positive Airway Pressure, CPAP, tracheal tube, Intubation kit, etc. |
| COVID diagnostics | Thermometer, rapid diagnostic test, antigen test, lateral flow assay, etc. |
| COVID vaccines | AD26.COV2.S, Sinovac, Sputnik V, etc. |
| All COVID searches | All of the above |

Key terms used for the MQM Glob web app’s COVID-19 shortcut queries

1. Personal Protective Equipment

((”Personal protective equipment” OR ”PPE” OR ”protective glasses” OR ”apron” OR ”n95” OR ”gowns” OR ”facemask” OR ”visor” OR ”gloves” OR ”goggles” OR ”respirator” OR ”KN95” OR ”face shield” OR ”mask”) OR ((”Medical devices for disease prevention”) AND (”COVID-19” OR ”COVID” OR ”SARS-CoV-2” OR ”Coronavirus” OR ”CV19” OR ”CV-19” OR ”SARS” OR ”CoV-2”)))

2. COVID-19 diagnostics

(”Thermometer” OR ((”coronavirus kit” OR ”RDT” OR ”covid test” OR ”lateral flow assay” OR ”test kit” OR ”LFA” OR ”COVID kit” OR ”Medical device for screening/diagnosis/monitoring” OR ”rapid diagnostic test” OR ”coronavirus test” OR ”antigen test” OR ”COVID-19 test” OR ”test cassette” OR ”In-vitro-diagnostic” OR ”cassette test” OR ”RT-PCR” OR ”IVD” OR ”testing kit” OR ”qPCR” OR ”antibody test” OR ”COVID-19 kit” OR ”PCR” OR ”polymerase chain reaction” OR ”ELISA”) AND (”COVID-19” OR ”COVID” OR ”SARS-CoV-2” OR ”Coronavirus” OR ”CV19” OR ”CV-19” OR ”SARS” OR ”CoV-2”)))

3. Sanitisers & disinfectants

”wipes” OR ”disinfectant” OR ”sanitizer” OR ”sanitizing” OR ”iodoform” OR ”sanitiser”

4. Vaccines

((”BNT162b2” OR ”BBIBP-CorV” OR ”Ad26.COV2.S” OR ”CoronaVac” OR ”Covishield” OR ”Ad5-nCoV” OR ”AZD1222” OR ”FBRI” OR ”Sputnik V” OR ”mRNA-1273” OR ”EpiVacCorona” OR ”Vero Cells” OR ”Covaxin”) OR ((”vaccine”) AND (”Barat Biotech” OR ”BioNTech” OR ”Johnson & Johnson” OR ”Pfizer” OR ”Oxford/AstraZeneca” OR ”Serum Institute of India” OR ”Sinopharm” OR ”Sinovac” OR ”Gamaleya” OR ”Moderna” OR ”Pfizer/BioNTech” OR ”CanSino” OR ”AstraZeneca” OR ”Oxford”)) OR ((”vaccine”) AND (”COVID-19” OR ”COVID” OR ”SARS-CoV-2” OR ”Coronavirus” OR ”CV19” OR ”CV-19” OR ”SARS” OR ”CoV-2”)))

5. Ventilation & oxygenation equipment and consumables

(”Continuous Positive Airway Pressure” OR ”Oxygen” OR ”nasal catheter ” OR ”CPAP” OR ”oximeter” OR ”positive end-expiratory pressure” OR ”PEEP” OR ”positive end expiratory pressure” OR ”bag-valve-mask” OR ”self-inflating bag” OR ”oropharyngeal catheter” OR ”BMV” OR ”nebulizer” OR ”tracheostomy tube” OR ”tracheal tube” OR ”ambu bag” OR ”ventilator” OR ”bag valve” OR ”nasal cannula” OR ”manual resuscitator” OR ”HEPA filter” OR ”endotracheal tube” OR ”air purifier” OR ”intubation kit”)

6. COVID-19 medicines

”tranilast” OR ”interleukin-2” OR ”INC424” OR ”TNKase” OR ”nitazoxanide” OR ”LY3832479” OR ”baloxavir” OR ”interleukin-7” OR ”Kineret” OR ”ritonavir” OR ”Crizanlizumab” OR ”Apixaban” OR ”cyclosporin” OR ”losartan” OR ”ATI-450” OR ”nitrogen monoxide” OR ”tirofiban” OR ”Ebselen” OR ”corbistadine” OR ”atorvastatin” OR ”Eicosapentaenoic” OR ”nitrite” OR ”Riamilovir” OR ”black cumin” OR ”NK-1R” OR ”Pemziviptadil” OR ”colchicine” OR ”Lithium” OR ”Vancomycin” OR ”Broncho-Vaxom” OR ”ramipril” OR ”Teicoplanin” OR ”tofacitinib” OR ”budesonide” OR ”Paracetamol” OR ”dipyridamole” OR ”levamisole” OR ”atovaquone” OR ”Senicapoc” OR ”covid drug” OR ”enoxaparin” OR ”Brequinar” OR ”povidone-iodine” OR ”levilimab” OR ”degarelix” OR ”LY3819253” OR ”Sofusbovir” OR ”masitinib” OR ”Omega-3” OR ”INM005” OR ”RBT-9” OR ”deferoxamine” OR ”canakinumab” OR ”Ramelteon” OR ”chlorpromazine” OR ”selinexor” OR ”Piclidenoson” OR ”DAS181” OR ”M5049” OR ”Ibudilast” OR ”CM4620-IE” OR ”GNS561” OR ”zanubrutinib” OR ”Cenicriviroc” OR ”sofosbovir” OR ”Trimethoprim” OR ”vadadustat” OR ”AVM0703” OR ”Rabeprazole” OR ”Moxifloxacin” OR ”cobicistat” OR ”BAT2020” OR ”ABX464” OR ”XAV-19” OR ”thalidomide” OR ”bamlanivimab” OR ”GX-19” OR ”corticosteroid” OR ”Tradipitant” OR ”cotrimoxazole” OR ”HuMax-Inflam” OR ”Apilimod” OR ”DUR-928” OR ”escin” OR ”PF06650833” OR ”octagam” OR ”Antroquinonol” OR ”pacritinib” OR ”Imatinib” OR ”ribavirin” OR ”ambrisentan” OR ”baricitinib” OR ”imatinib” OR ”CD24Fc” OR ”Sulodexide” OR ”AlloStim” OR ”DFV890” OR ”Emapalumab” OR ”sitagliptin” OR ”Metformin” OR ”prednisone” OR ”ulinastatin” OR ”naltrexone” OR ”abidor” OR ”niclosamide” OR ”BIO101” OR ”GS-441524” OR ”argatroban” OR ”Leukine” OR ”xiyanping” OR ”peginterferon” OR ”pembrolizumab” OR ”HuMax” OR ”Lambda” OR ”dornase” OR ”Itraconazole” OR ”telemedicine” OR ”Adenosine” OR ”nirmatrelvir” OR ”Curosurf” OR ”clarithromycin” OR ”bromhexine” OR ”Xpovio” OR ”ebastine” OR ”amoxicillin/clavulanate” OR ”PD-1 mAb” OR ”EPA” OR ”oseltamivir” OR ”Betamethasone” OR ”favipiravir” OR ”mefloquine” OR ”bismuth” OR ”CM4620” OR ”ifenprodil” OR ”Levofloxacin” OR ”REGN10987” OR ”Candesartan” OR ”secukinumab” OR ”Trihexyphenidyl” OR ”Daclatasvir” OR ”pinavir” OR ”tocilizumab” OR ”co-amoxiclav” OR ”EG-HPCP-03a” OR ”hydroxychloroquine” OR ”Polyoxidonium” OR ”STI-5656” OR ”Artesunate” OR ”triazavirine” OR ”Disulfiram” OR ”cholecalciferol” OR ”INO-4800” OR ”PG1” OR ”zinc” OR ”oxytocin” OR ”gimsilumab” OR ”suramin” OR ”rhG-CSF” OR ”desferoxamine” OR ”TD-0903” OR ”OM-85” OR ”Bucillamine” OR ”pirfenidone” OR ”Acetaminophen” OR ”adamumab” OR ”sulfamethoxazole” OR ”BI 764198” OR ”RPH-104” OR ”COVID-19 drug” OR ”alpha lipoic” OR ”almitrine” OR ”melphalan” OR ”dapagliflozin” OR ”NBTNM108” OR ”TMJ2” OR ”Icosapent” OR ”Ceftriaxone” OR ”isoprinosine” OR ”IMU838” OR ”tridecactide” OR ”chloroquine” OR ”CSL324” OR ”Lian Hua Qing Weng” OR ”Kevzara” OR ”valsartan” OR ”meplazumab” OR ”Namilumab” OR ”Prednisolone” OR ”sargramostim” OR ”estradiol” OR ”cyclosporine” OR ”Aprepitant” OR ”silymarin” OR ”linagliptin” OR ”Noscapine” OR ”Gemtuzumab” OR ”methylprednisolone” OR ”fluvoxamine” OR ”Coroquard” OR ”mavrilimumab” OR ”anakinra” OR ”ozanimod” OR ”mepolizumab” OR ”acetylsalicylic” OR ”darunavir” OR ”novaferon” OR ”YinHu QingWen” OR ”OM85” OR ”camrelizumab” OR ”Cosentyx” OR ”estrogen” OR ”dexmedetomidine” OR ”LL-37” OR ”Dantonic” OR ”rivaroxaban” OR ”adalimumab” OR ”apremilast” OR ”polyinosinic-polycytidylic” OR ”farpiravir” OR ”montelukast” OR ”Ibuprofen” OR ”IFX-1” OR ”Iodine” OR ”Molnupiravir” OR ”Pioglitazone” OR ”verapamil” OR ”Rapamycin” OR ”Brexanolone” OR ”Eltrombopag” OR ”ravulizumab” OR ”hydrocortisone” OR ”auxora” OR ”tinzaparin” OR ”Vascepa” OR ”omalizumab” OR ”Tybost” OR ”Actemra” OR ”dociparastat” OR ”NA-831” OR ”ascorbic acid” OR ”MAS825” OR ”C21” OR ”RoActemra” OR ”eculizumab” OR ”Bivalirudin” OR ”povidon-iodine” OR ”ivermectin” OR ”Pamrevlumab” OR ”danoprevir” OR ”Neurokinin” OR ”sirolimus” OR ”Fostamatinib” OR ”resveratrol” OR ”Icatibant” OR ”bromelain” OR ”dexamethasone” OR ”TJ003234” OR ”iloprost” OR ”tacrolimus” OR ”astegolimab” OR ”interferon” OR ”plitidepsin” OR ”metenkefalin” OR ”azoximer” OR ”lopinavir” OR ”Tazobactam” OR ”carrimycin” OR ”CM-4620” OR ”CYT107” OR ”Heparin” OR ”PyronaridineArtesunate” OR ”Itolizumab” OR ”zilucoplan” OR ”oxpentifylline” OR ”AT-001” OR ”Abivertinib” OR ”doxycycline” OR ”Nigella Sativa” OR ”AZD1222” OR ”leronlimab” OR ”Enalapril” OR ”nangibotide” OR ”Piperacillin” OR ”bevacizumab” OR ”lactoferrin” OR ”UTTR1147A” OR ”Caesalpinia spinosa” OR ”mometasone” OR ”hydroxychloroquin” OR ”Febuxostat” OR ”lanadelumab” OR ”Thymalfasin” OR ”huaier extract” OR ”Levoflozacin” OR ”Pentoxifylline” OR ”tozumab” OR ”NP-120” OR ”Alvelestat” OR ”captopril” OR ”merimepodib” OR ”Iota-Carrageenan” OR ”Lianhua Qingwen” OR ”GLS-1200” OR ”aescinate” OR ”tranexamic” OR ”Ledipasvir” OR ”ISIS 721744” OR ”procalcitonin” OR ”SNDX-6352” OR ”sirukumab” OR ”Enzalutamide” OR ”carriomycin” OR ”amphotericin” OR ”bemiparin” OR ”T89” OR ”Spironolactone” OR ”sotrovimab” OR ”fingolimod” OR ”aspirin” OR ”Remdesivir” OR ”TJM2” OR ”pyridostigmine” OR ”Prolastin” OR ”EC-18” OR ”poractant” OR ”isotretinoin” OR ”telmisartan” OR ”lenzilumab” OR ”avdoralimab” OR ”duvelisib” OR ”BIO 300” OR ”bicalutamide” OR ”Ilaris” OR ”atlizumab” OR ”desferrioxamine” OR ”LB1148” OR ”Regkirona” OR ”vitamin D3” OR ”Clopidogrel” OR ”CD24” OR ”tetrandrine” OR ”Lansoprazole” OR ”Ruconest” OR ”amoxicillin” OR ”Trifluoperazine” OR ”Ganovo” OR ”nitric Oxide” OR ”chlorine dioxide” OR ”olokizumab” OR ”lucinactant” OR ”galidesivir” OR ”TXA127” OR ”Maraviroc” OR ”conestat” OR ”CA S001” OR ”vazegepant” OR ”REGN10933” OR ”Propranolol” OR ”Viagra” OR ”Fisetin” OR ”Ronapreve” OR ”Previfenon” OR ”omega 3” OR ”regdanvimab” OR ”thymosin” OR ”Prasugrel” OR ”retinoic acid” OR ”Ceftaroline” OR ”sevoflurane” OR ”amoxicillin/clavulanic acid” OR ”oestrogen” OR ”leflunomide” OR ”virazole” OR ”PLN-74809” OR ”ATYR1923” OR ”Olumiant” OR ”dalargin” OR ”Alinia” OR ”methotrexate” OR ”dapansutrile” OR ”artemisinin” OR ”ibrutinib” OR ”aescin” OR ”CERC-002” OR ”fludase” OR ”isoflurane” OR ”XPro1595” OR ”LY-CoV555” OR ”CAS0001” OR ”immunoglobulin” OR ”nafamostat” OR ”Crocetinate” OR ”Diphenhydramine” OR ”BIO 101” OR ”AZD1656” OR ”PTC299” OR ”amodiaquine” OR ”casirivimab” OR ”BGB-DXP593” OR ”opaganib” OR ”melatonin” OR ”huaier granule” OR ”HuMax-IL8” OR ”Paxlovid” OR ”famotidine” OR ”GLS1027” OR ”Trimodulin” OR ”tenofovir” OR ”Primaquine” OR ”AMY-101” OR ”covid medicine” OR ”umifenovir” OR ”EDP1815” OR ”Lagevrio” OR ”Vitamin B12” OR ”Gamunex-C” OR ”Bardoxolone” OR ”AstroStem-V” OR ”LAU-7b” OR ”Vitamin E” OR ”Vitamin B” OR ”RTB101” OR ”COVID-19 medicine” OR ”curcumin” OR ”fondaparinux” OR ”Edoxaban” OR ”L-Citrulline” OR ”ciclesonide” OR ”azithromycin” OR ”remdesivir” OR ”Diltiazem” OR ”Methylene blue” OR ”clazakizumab” OR ”BCX4430” OR ”Pyronaridine” OR ”Quercetin” OR ”Toremifene” OR ”COVI-AMG” OR ”etoposide” OR ”DWJ1248” OR ”defibrotide” OR ”AT-527” OR ”prazosin” OR ”triazavirin” OR ”BIO300” OR ”Ensifentrine” OR ”coronavirus medicine” OR ”Anti-IL-8” OR ”dihydroartemisinin” OR ”vitamin c” OR ”25-hydroxyvitamin D3” OR ”coronavirus drug” OR ”formoterol” OR ”indomethacin” OR ”Rayaldee” OR ”ciclosporin” OR ”naproxen” OR ”fluoxetine” OR ”Infliximab” OR ”Tenecteplase” OR ”ruxolitinib” OR ”Molgramostim” OR ”vitamin D” OR ”simvastatin” OR ”alteplase” OR ”sildenafil” OR ”isoquercetin” OR ”GC4419” OR ”ketamine” OR ”Razuprotafib” OR ”camostat” OR ”Arbidol” OR ”Montmorrillonite” OR ”acalabrutinib” OR ”nivolumab” OR ”aviptadil” OR ”PUL-042” OR ”diammonium” OR ”Clevudine” OR ”nitrogen oxide” OR ”BMS-986253” OR ”siltuximab” OR ”interleukin 2” OR ”jakotinib” OR ”nintedanib” OR ”Axatilimab” OR ”garadacimab” OR ”Treamid” OR ”ASC09” OR ”emtricitabine” OR ”LY-CoV016” OR ”Pulmozyme” OR ”Prostaglandin” OR ”ciclosporine” OR ”hydrogen peroxide” OR ”sarilumab” OR ”Losmapimod” OR ”azvudine” OR ”BLD-2660” OR ”EIDD-2801” OR ”MSTT1041A” OR ”Desidustat” OR ”abidole” OR ”omeprazole” OR ”progesterone” OR ”Decitabine” OR ”tocopherol” OR ”berberine” OR ”Xevudy” OR ”APL-9” OR ”colomycin” OR ”XC221” OR ”amiodarone” OR ”lenalidomide” OR ”imdevimab” OR ”ixekizumab” OR ”VentaProst” OR ”acetylcysteine” OR ”LY3127804” OR ”Atazanavir” OR ”TL-895” OR ”dalteparin” OR ”Thimerosal” OR ”Xue-Bi-Jing” OR ”GC376” OR ”Angiotensin” OR ”gs441542” OR ”Risankizumab” OR ”co-trimoxazole”) OR ((”Medicine” OR ”Plasma” OR ”Treatment” OR ”Medication” OR ”Monoclonal antibodies” OR ”Antibody therapy” OR ”Antibody cocktail”) AND (”COVID-19” OR ”COVID” OR ”SARS-CoV-2” OR ”Coronavirus” OR ”CV19” OR ”CV-19” OR ”SARS” OR ”CoV-2”
