# Supplementary material for: The Medicine Quality Monitoring Globe: A public health intelligence system for collecting and curating reports of substandard and falsified medical products from publicly available Internet news media
Source: PLOS Digit Health. 2026 Sep 15;5(9):e0001648. doi: 10.1371/journal.pdig.0001648 (PMC13577332; doi:10.1371/journal.pdig.0001648)
Supplement: Supplementary file 3 [file pdig.0001648.s003.docx]

Supplementary Materials 3

**Information sources for capture by direct RSS**

The sources are webpages from selected MRA, specific newspapers not referenced in Google News that previously published on SF medical products, and various international organisations. RSS feeds are filtered based on a set of inclusion terms related to medicine quality and exclusion terms related to common false-positive topics.

**English**

US Food and Drug Administration (various newsletters)

Food and Drug Administration of the Philippines

Hindustan Times

Institute of Research against Counterfeit Medicines (IRACM)

MedicalDialogues.com, Pharmaceutical News

Ministry of Health, Belize

Modern Ghana RSS

National Drug Authority, Uganda

SecuringIndustry.com, Pharmaceuticals

US Customs and Border Protection News

Zambia Medicines Regulatory Authority

**French**

ANSM France

CAMEG, Burkina Faso

Centre National de Pharmacovigilance et de Matériovigilance, Algérie

Direction de la Pharmacie et du Médicament, Républic du Sénégal

Direction générale de la concurrence, de la consommation et de la répression des fraudes (DGCCRF), France

Fondation Chirac, France

IRACM

Ministère des Solidarités et de la Santé, France

Ministère Economique Actualités Generales, France

**Spanish**

Agencia Española de Medicamentos y Productos Sanitarios

Ministerio de Gobierno, Ecuador

Ministerio de Salud Pública, Ecuador

Ministerio de Salud Pública, Republica Dominicana
