# Supplementary material for: The Medicine Quality Monitoring Globe: A public health intelligence system for collecting and curating reports of substandard and falsified medical products from publicly available Internet news media
Source: PLOS Digit Health. 2026 Sep 15;5(9):e0001648. doi: 10.1371/journal.pdig.0001648 (PMC13577332; doi:10.1371/journal.pdig.0001648)
Supplement: Supplementary file 4 [file pdig.0001648.s004.docx]

Supplementary Materials 4

**Examples of search queries illustrating the flexibility of the MQM Globe web app search function**

| **Search String** | **Meaning** |
| --- | --- |
| Uganda | Searches reports for the name Uganda |
| Africa | Searches reports for the name Africa. On indexing any reports containing the name of an African country would have the name Africa added to the index. |
| Afri* | The wildcard ‘*’ character is used to match zero or more characters. For example, Afri* will find the name Africa as well as Afrikaans. |
| Afica~ | The query parser will complete a fuzzy search on the term ‘afica’ and will typically find Africa |
| Africa AND Asia | Returns only those reports that contain both Africa AND Asia. N.B. the ‘AND’ must be in uppercase to be interpreted as a logic element. |
| snippet:impounded | Searches for the word ‘impounded’ only in the snippet of all the reports. |
| Vaccines NOT China | Search for all reports about vaccines that do not include details of a place in China. N.B. the ‘NOT’ needs to be in uppercase to be identified as a logic element. |
| benzodiazepine | The system looks for ‘benzodiazepine’ within the ATC table and will return any reports that contain medicines with ATC codes belonging to lower-level codes. For example, ‘benzodiazepine,’ found in the ATC classification as ‘benzodiazepine derivatives’ with ATC code “N05BA” will return reports containing ‘diazepam’ (N05BA01), ‘chlordiazepoxide’ (N05BA02), ‘medazepam’ (N05BA03), ‘oxazepam’ (N05BA04) etc. |
| “Central Africa” | Returns reports for countries in Central Africa |
| Central Africa | Returns reports for countries in Central Africa and for countries in Africa and for the word ‘Central’, e.g., Central Asia, etc. |
| Note: the search engine also performs stemming to remove plurals. | |
