# Supplementary material for: The Medicine Quality Monitoring Globe: A public health intelligence system for collecting and curating reports of substandard and falsified medical products from publicly available Internet news media
Source: PLOS Digit Health. 2026 Sep 15;5(9):e0001648. doi: 10.1371/journal.pdig.0001648 (PMC13577332; doi:10.1371/journal.pdig.0001648)
Supplement: Supplementary file 5 [file pdig.0001648.s005.docx]

Supplementary Materials 5

**Number of unique users of the Medicine Quality Monitoring Globe per year per country (top 10)**

| Country | 2020 | 2021 | 2022 | 2023 | 2024 |
| --- | --- | --- | --- | --- | --- |
| United States of America | 274 | 238 | 185 | 244 | 128 |
| United Kingdom of Great Britain and Northern Ireland | 111 | 134 | 115 | 86 | 79 |
| France | 93 | 50 | 30 | 20 | 14 |
| Germany | 20 | 22 | 16 | 58 | 42 |
| India | 11 | 23 | 19 | 13 | 43 |
| Switzerland | 32 | 33 | 10 | 10 | 20 |
| Niger | 67 | 35 | 1 | 0 | 1 |
| China | 2 | 16 | 21 | 19 | 10 |
| Belgium | 18 | 13 | 9 | 11 | 7 |
| Netherlands | 20 | 13 | 12 | 7 | 5 |
