# Supplementary material for: The Medicine Quality Monitoring Globe: A public health intelligence system for collecting and curating reports of substandard and falsified medical products from publicly available Internet news media
Source: PLOS Digit Health. 2026 Sep 15;5(9):e0001648. doi: 10.1371/journal.pdig.0001648 (PMC13577332; doi:10.1371/journal.pdig.0001648)
Supplement: Supplementary file 6 [file pdig.0001648.s006.docx]

Supplemental Materials 6

**Medicine Quality Monitoring Globe disclaimer and caveats**

[displayed on the Globe website]

MQ Globe Takedown Statement

Our MQ globe is subject to a take down policy. If we are contacted by a potential rights-holder who objects to the presence of material on-line, we will remove the material in question from our website until we have been able to assess the case. Where material is removed for valid reasons of copyright, its removal will be considered as lasting until copyright in the material expires, or until the rights-holder agrees that the material can be reinstated.

To contact us about removal of material, please email us at medicinequality@iddo.org.

The System

This system aggregates, tabulates and maps publicly available information on the quality of medical products globally from the non-peer reviewed lay literature, including online newspapers, websites and blogs. The information is extracted from these resources and responsibility for the accuracy of these data and their interpretation rests with the primary resource. As the Essential Medicines Surveyor represents data from the scientific literature we expect that it will be more reliable and trustworthy than those data represented in the Medicines Quality Monitoring Globe. The information in the Medicines Quality Monitoring Globe usually will not have scientific confirmation. Therefore, please interpret the information in the Globe with the greatest caution. We regard such reports as early warnings of potential problems that need further investigation, if not already conducted.

The data are geographically heterogeneous and an important caveat is that no/few news or reports from a country or area does not imply that medical product quality there is good but that there are no/few accessible data from that country or area. Similarly, many news and reports of poor quality medical products in a country does not imply that medical product quality there is universally grave in comparison to elsewhere. Countries with many news reports should be lauded for facilitating such reporting. In addition, although we endeavour to include published corrections of previously published news we cannot guarantee that these are included.

Importantly there is often confusion in the lay literature between the terms falsified, substandard, spurious, fraudulent, degraded and counterfeit. Please interpret the use of these terms with great caution. See the [WHO recommended terms](https://www.who.int/news-room/fact-sheets/detail/substandard-and-falsified-medical-products) [https://www.who.int/news-room/fact-sheets/detail/substandard-and-falsified-medical-products].

If you notice any errors or omissions please contact us at medicinequality@iddo.org.

University of Oxford

The information contained in this website is for general information purposes only. The information is provided by the University of Oxford and while we endeavour to keep the information up to date and correct, we make no representations or warranties of any kind, express or implied, about the completeness, accuracy, reliability, suitability or availability with respect to the website or the information or related graphics contained on the website for any purpose. Any reliance you place on such information is therefore strictly at your own risk and based on the original news report. In no event will we be liable for any loss or damage whatsoever arising out of, or in connection with, the use of this website including without limitation, indirect or consequential loss or damage, loss of data or profits, or loss of business. Through this website you are able to link to other websites which are not under the control of the University of Oxford. We have no control over the nature, content and availability of those sites. The inclusion of any links does not necessarily imply a recommendation or endorse the views expressed within them. Every effort is made to keep the website up and running smoothly. However, the University of Oxford takes no responsibility for, and will not be liable for, the website being temporarily unavailable due to technical issues beyond our control.

Please also see [IDDO's terms of use](https://www.iddo.org/terms-of-use) [https://www.iddo.org/terms-of-use].
