# Supplementary material for: The Medicine Quality Monitoring Globe: A public health intelligence system for collecting and curating reports of substandard and falsified medical products from publicly available Internet news media
Source: PLOS Digit Health. 2026 Sep 15;5(9):e0001648. doi: 10.1371/journal.pdig.0001648 (PMC13577332; doi:10.1371/journal.pdig.0001648)
Supplement: Supplementary file 7 [file pdig.0001648.s007.docx]

**Minor Revision Comments (Suggestions for Improvement):**

1. The manuscript is well written and presents an intriguing digital monitoring system. However, we could enhance the clarity, robustness, and future applicability of the proposed framework by improving a few aspects.
2. The automated relevance classifier shows a relatively low precision (Precision: 37%, Recall: 90%, F1 Score: 52%) compared to its recall. Although the high recall guarantees the capture of relevant reports, the lower precision could potentially lead to a significant number of false positives. The authors may consider discussing possible improvements, such as the use of more advanced natural language processing models (e.g., transformer-based approaches) to improve classification accuracy.
3. The performance of the georeferencing component appears relatively limited (Precision: 30%, Recall: 28%, F1 score: 29%). Since accurate location identification is critical for mapping incidents related to medicine quality, the authors may consider discussing potential improvements in geographic entity recognition methods.
4. The system relies heavily on publicly available news reports for identifying medicine quality incidents. Your study already quotes, "News data often remains unverified and should be interpreted cautiously." Since such reports may sometimes contain incomplete or unverified information, it would be beneficial for the authors to discuss possible validation mechanisms or cross-verification strategies.
5. The current system appears to depend significantly on specific news aggregation platforms. The authors may briefly discuss the potential limitations of such dependency and whether alternative data sources or APIs could be integrated in future versions of the system.
6. Although the system supports multiple languages, expanding the language coverage could further improve global monitoring capability. The authors may consider mentioning plans for integrating additional languages in future updates. As you have mentioned in your study on Indian fake news, India is the most populous country. Therefore, you might consider including Hindi and several other languages.
7. The manuscript indicates that duplicate reports across different languages may not always be automatically identified. Introducing cross-language similarity detection techniques could improve data consistency and reduce redundant entries.
8. The current workflow includes manual curation steps for validating and annotating reports. While this improves data quality, the authors may discuss possible semi-automated approaches that could improve scalability when processing large volumes of reports.
9. Future Real-Time Alert Capability: The system primarily functions as a monitoring and visualization platform. The usefulness of the system could be further enhanced by incorporating automated real-time alert mechanisms that notify relevant authorities when high-risk incidents are detected.
