# Supplementary material for: The Medicine Quality Monitoring Globe: A public health intelligence system for collecting and curating reports of substandard and falsified medical products from publicly available Internet news media
Source: PLOS Digit Health. 2026 Sep 15;5(9):e0001648. doi: 10.1371/journal.pdig.0001648 (PMC13577332; doi:10.1371/journal.pdig.0001648)
Supplement: Supplementary file 8 [file pdig.0001648.s008.docx]

**Minor Revision Comments (Suggestions for Improvement):**

Reviewer #1 comments:

1. **The manuscript is well written and presents an intriguing digital monitoring system. However, we could enhance the clarity, robustness, and future applicability of the proposed framework by improving a few aspects.**

We thank the reviewers for their feedback on the manuscript and are pleased to make the changes as shown in the revised document.

2. **The automated relevance classifier shows a relatively low precision (Precision: 37%, Recall: 90%, F1 Score: 52%) compared to its recall. Although the high recall guarantees the capture of relevant reports, the lower precision could potentially lead to a significant number of false positives. The authors may consider discussing possible improvements, such as the use of more advanced natural language processing models (e.g., transformer-based approaches) to improve classification accuracy.**

Given the low precision of the relevance classifier (37% for English-language content), curators currently need to review a large volume of false positives. Based on our preliminary work applying LLM tools, we believe we can use them to increase precision substantially. We also believe we can improve the performance of current georeferencing automation with these tools, and extend the automation to languages beyond English.

We have addressed this comment on page 22, line 537 of the revised manuscript.

3. **The performance of the georeferencing component appears relatively limited (Precision: 30%, Recall: 28%, F1 score: 29%). Since accurate location identification is critical for mapping incidents related to medicine quality, the authors may consider discussing potential improvements in geographic entity recognition methods.**

We agree that this performance level is low. At this phase we are seeing promising results from initial work with large language models to perform this task. Even with advanced techniques, the task is difficult to perform given its open-ended nature. There are many millions of possible geographic categories, including differing levels of geographic resolution, and differing semantic roles that geographic categories can serve (for example, the location of a law enforcement action, versus the origin of seized product). We have added reference to our ongoing work, not yet ready for publication, on page 22, line 524 of the revised manuscript.

4. **The system relies heavily on publicly available news reports for identifying medicine quality incidents. Your study already quotes, "News data often remains unverified and should be interpreted cautiously." Since such reports may sometimes contain incomplete or unverified information, it would be beneficial for the authors to discuss possible validation mechanisms or cross-verification strategies.**

We thank the reviewer for this important observation. We acknowledge that reliance on news reports introduces inherent limitations regarding accuracy and completeness. Currently the authors are working on 2 aspects of this point, as part of separate research work, outside the scope of the submitted article.

- To strengthen the use of news media data, the researchers suggest the development of an intelligence grading system adapted from law enforcement.
- Further research could investigate cross-validation with existing reference standards. The authors are assessing the performance of the MQM Globe (sensitivity and timeliness) compared to the WHO Medical Product Alerts (official WHO announcements). The study is completed and will be submitted for publication soon

These aspects have been added in the Discussion, page 21, line 497.

5. **The current system appears to depend significantly on specific news aggregation platforms. The authors may briefly discuss the potential limitations of such dependency and whether alternative data sources or APIs could be integrated in future versions of the system.**

We have added to the discussion section on this point; please see page 22, line 555 of the revised manuscript.

6. **Although the system supports multiple languages, expanding the language coverage could further improve global monitoring capability. The authors may consider mentioning plans for integrating additional languages in future updates. As you have mentioned in your study on Indian fake news, India is the most populous country. Therefore, you might consider including Hindi and several other languages.**

We thank the reviewer for this comment and agree with this suggestion. This aspect has been addressed in the Discussion section (page 23, lines 548-550), where we acknowledge that expanding language coverage represents an important next step to improve the detection of SF product issues in different geographical areas and to reduce inclusion bias.

We would like to add more languages to the system. However, we have been primarily constrained by the need for funding for analysts with expertise in public health and pharmaceutical products who are fluent in these additional languages.

7. **The manuscript indicates that duplicate reports across different languages may not always be automatically identified. Introducing cross-language similarity detection techniques could improve data consistency and reduce redundant entries.**

We thank the reviewer for identifying this important technical challenge. Currently different languages should be approached as different information streams. On top of increasing coverage and representability, by including multiple languages we hoped to provide information to users speaking different languages. When we used data from multiple languages for more in-depth analysis (ongoing study by the authors), we indeed needed to account for duplication between languages. We fully agree that deduplication across languages would be a valuable improvement. We are currently investigating cross-language deduplication using LLMs.

Please see the related update to the manuscript discussion, page 23, line 551.

8. **The current workflow includes manual curation steps for validating and annotating reports. While this improves data quality, the authors may discuss possible semi-automated approaches that could improve scalability when processing large volumes of reports.**

We appreciate this valuable comment. We agree that manual curation presents scalability challenges. We try to continually improve the hybrid approach with the aim to have an automated filtering and characterisation but with human validation.

We are working actively on improving automation. (as described in the methods under section c, Automated characterisation, page 8, line 195). Furthermore, large language models are becoming more powerful, and we are actively working to integrate more AI-based processes. Please see the Discussion section, page 22, lines 530-542.

We are committed to maintaining a human-in-the-loop system. We deliberately adopt a hybrid system to ensure accuracy and contextual interpretation.

9. **Future Real-Time Alert Capability: The system primarily functions as a monitoring and visualization platform. The usefulness of the system could be further enhanced by incorporating automated real-time alert mechanisms that notify relevant authorities when high-risk incidents are detected.**

We thank the reviewer for this forward-looking suggestion. We agree that integrating real-time alert capabilities would significantly enhance the practical value of the system. The back-end development of the alerting system is ongoing, and we liaise with end-users working in public health and law enforcement (e.g. WHO, UNODC, or Europol). In parallel we are working on a grading system to define the threshold for dissemination. Please see discussion page 21, lines 497-500.

Reviewer #2 comments:

1. **The performance is not satisfactory, with the reported F1-score being only 52%.**

We appreciate this note from the reviewer; in fact, we are also not satisfied with the performance of the machine learning system, in particular its precision of 37% on English-language content and lower on French-language. While we are not able to increase the precision for the present article, we are currently using transformer-based large language models in combination with our existing machine learning system to improve the precision. Using the systems in combination, as opposed to using the LLM exclusively, allows for reduced cost and faster speed of processing, especially given that the level of true positives is extremely low. As indicated in Table 3, the number of relevant articles we collect is around 2% of the total volume (across all languages) with the number of distinct, relevant articles being close to 1% of the volume. Thus, though the precision is low, the system does offer a significant advantage over a purely manual approach. We have updated the Discussion section, on page 22, lines 530 – 542, to indicate our work to improve the system using LLMs.

2. **"The extent of the problem is difficult to measure as most often the quality of a given medical product cannot be determined without specialised equipment and expertise in the relatively few reference laboratories globally." Given this, how can the Internet and electronic media be relied upon for accurate information about medical products? Please provide a clear rationale and supporting evidence for the study’s aims and practical implications.**

We appreciate this insightful comment. Our approach does not aim to assess the quality of medical products but rather identify signals indicative of potential quality issues. The online media source can provide an early indication of risks that may not yet be captured through traditional surveillance systems.

Due to the lack of data in the field regarding poor-quality medical products, we want to improve use of all available data to support risk-based surveillance. This approach is supported by past success of digital surveillance in the context of food fraud and diseases. Since the launch of the MQM Globe, entities like UNODC (1) or USP (2) have been using the early warning signals in their work. We have clarified the revised manuscript in the introduction (page 4-5, lines 109-111) and in the discussion (pages 21, lines 483-485 and lines 497-500).

(1) United Nations Office on Drugs and Crime. Trafficking in medical products in sahel. Transnational Organized Crime Threat Assessment on the Sahel: United Nations Office on Drugs and Crime, 2023:28.

(2) Borse NN, Cha J, Chase CG, et al. Responding to the surge of substandard and falsified health products triggered by the Covid-19 pandemic. In: (USP) UP, ed. United States of America: US Pharmacopeia (USP), 2021:1-11.

3. **how did the authors ensure data representativeness and reliability?**

We acknowledge that reliance on news reports from the lay press introduces inherent limitations regarding accuracy and completeness. We refer to the disclaimer and caveats section on the MQM Globe website (<https://www.iddo.org/medicine-quality-monitoring-globe-disclaimer-and-caveats>). We have now added the disclaimer as supplementary material; please see Medicine Quality Monitoring Globe disclaimer and caveats on page 14 of the Supporting Information document.

**Reliability**

- The lay press information on the MQM Globe should complement existing surveillance data to support risk-based surveillance. Because of the limitations with lay press, the MQM Globe also has a tab for regulatory alerts (manuscript: Methods, pages 13-14, from lines 331). The Globe users can thus also find validated information from regulators who are actively distributing and publicly sharing this information.
- To strengthen the use of news media data, we are developing an intelligence grading system adapted from law enforcement to mitigate the impact of reports that are less reliable (manuscript: discussion, page 21, lines 497-500).

**Representativeness.**

The MQM Globe is not intended to be representative of SF related issues, rather, to detect (early) signals. It is hypothesis generating and a tool to support risk-based surveillance. By including multiple languages, we try, however, to improve the representativeness, and we intend to extend the number of languages. The authors are finalising a separate study on the performance of the MQM Globe in capturing the WHO Medical Product Alerts (official WHO announcements). It shows that the sensitivity of the MQM Globe was limited when using one language but was greatly improved when combining languages. The study is completed and will be submitted for publication soon. Further research could compare the reports in the MQM Globe with national medicines regulatory authorities that publish this information. This aspect has been added to the revised manuscript’s Discussion, page 21, lines 487-497.

4. **On what basis was the search engine selected?**

We use several search engines that were selected based on coverage, cost, and availability. We have added this to the Discussion of the revised manuscript; please see page 23, line 555.

5. **How was fake news identified and verified?**

We thank the reviewer for raising the important point on fake news (questions 5 and 6). We recognize that fake news might be recorded in the Globe. Our trained analysts are aware of the risk and when a fake news or publisher is identified, articles are removed from the Globe (‘BNN Breaking’ was recently identified as using AI tools to paraphrase news content that had led in the past to wrong information being published). We are developing an intelligence grading system adapted from law enforcement to mitigate the impact of reports that are less reliable which may support the identification of fake news (manuscript: Discussion, page 21, line 497).

A disclaimer is available on the Globe webpage. It informs the users that “The information in the MQM Globe usually will not have scientific confirmation. Therefore, please interpret the information in the MQM Globe with the greatest caution.” We regard such reports as early warnings of potential problems that need further investigation, if not already conducted. The Globe disclaimer has been added to the supplementary materials (see question 3)

**What measures were taken to assess the accuracy of fake news detection?**

If fake news is suspected, we investigate the case online and discuss among the team the action to be taken. However, it is difficult to confirm whether news is fake or not depending on the situation.

6. **Please provide details of the technology/algorithm/method of the relevance classifier and related detection.**

Please see citation 14 (cited on page 9 line 214 of the revised manuscript); this article details the algorithms we are using to compile training data into a model and then compute an indicator score for novel content using the model. We have implemented the algorithms in Python, using MongoDB to store and retrieve the model.

7. **Why were different languages included in the study?**

We thank the reviewer for raising this important point. In many countries, lay press articles are published only in local languages, hence the use of different languages, to allow better geographical coverage.

Researchers working on disease surveillance showed that combining multiple languages improves performance (3, 4). We are currently finalising a study that shows that the sensitivity of the MQM Globe was greatly improved when combining languages (publication forthcoming).

(3) Barboza P, Vaillant L, Le Strat Y, et al. Factors influencing performance of internet-based biosurveillance systems used in epidemic intelligence for early detection of infectious diseases outbreaks. PLoS One 2014;9(3):e90536. doi: 10.1371/journal.pone.0090536 [published Online First: 20140305]

(4) Sulaiman FB, Yanti NKS, Lesmanawati DAS, et al. Language-specific gaps in identifying early epidemic signals – a case study of the Malay language. Global Biosecurity 2019;1 doi: 10.31646/gbio.33

8. **Were any socio-cultural perspectives considered during the selection or analysis?**

Yes—we have employed native language analysts with understanding of the online media and of the local medical product context in the target language. Search terms were not simply translated; the analysts also identified synonyms commonly used by journalists/lay people. These analysts are co-authors of the article.

9. **How many data analysts were involved in the labelling process?**

For each language, one dedicated analyst conducted the labelling. Since the start of the MQM Globe system, there has been some (but limited) staff turnover, as is typical in long-term projects. All changes occurred within the context of a standardised labelling protocol (available on request).

Please see the Methods section of the manuscript, page 10, line 249-250.

10. **What was the level of agreement between analysts, and how was the labelling validated?**

We have conducted a blinded inter-rater agreement study for the English-language relevance classification task; please see results on page 17, line 394 of the revised manuscript.

11. **For Table 4: How were the reports validated to ensure they reliably described the medical products?**

We thank the reviewer for this important question. Whether a product was falsified, substandard or degraded, is often subject to interpretation. Journalists tend to use a variety of terms to describe poor quality products (counterfeit, spurious, falsified, fake etc) without definition of the terms being used, and they often seek sensationalism over accurateness of terms. The analysts are trained to look for clues to guide the choice of label. However, the MQM Globe does not independently validate the information in the online news media reports. The responsibility for the accuracy of the data rests with the original reporting entity.

To clarify this point for readers, we changed the text in the discussion to acknowledge that limitation, and we added the MQM Globe disclaimer and caveats, which gives more background on the limitations of the data as a supplement (<https://www.iddo.org/medicine-quality-monitoring-globe-disclaimer-and-caveats>). Please see Discussion, page 21, line 487-489, and Supporting Information, page 14.
