# Supplementary material for: The Medicine Quality Monitoring Globe: A public health intelligence system for collecting and curating reports of substandard and falsified medical products from publicly available Internet news media
Source: PLOS Digit Health. 2026 Sep 15;5(9):e0001648. doi: 10.1371/journal.pdig.0001648 (PMC13577332; doi:10.1371/journal.pdig.0001648)
Supplement: Peer review history

# Peer review: Decision Letter 0

**DOI:** 10.1371/journal.pdig.0001648.r001

**Authors:** Jasmit Shah, Laura Sbaffi

24 Mar 2026

Response to Reviewers Revised Manuscript with Track Changes Manuscript **Journal Requirements:**

1. Please provide a detailed online Financial Disclosure statement. This is published with the article. It must therefore be completed in full sentences and contain the exact wording you wish to be published.

a) Please clarify all sources of financial support for your study. List the grants, grant numbers, and organizations that funded your study, including funding received from your institution. Please note that suppliers of material support, including research materials, should be recognized in the Acknowledgements section rather than in the Financial Disclosure.

b) State the initials, alongside each funding source, of each author to receive each grant. For example: “This work was supported by the National Institutes of Health (####### to AM; ###### to CJ) and the National Science Foundation (###### to AM).”

c) State what role the funders took in the study. If the funders had no role in your study, please state: “The funders had no role in study design, data collection and analysis, decision to publish, or preparation of the manuscript.”

For more information, please go to our submission guidelines:

https://journals.plos.org/digitalhealth/s/submission-guidelines#loc-financial-disclosure-statement

2. Please ensure that the funders and grant numbers match between the Financial Disclosure field and the Funding Information tab in your submission form. Note that the funders must be provided in the same order in both places as well.

3. Please provide separate main figure files in .tif or .eps format only and ensure that all files are under our size limit of 10MB.

For more information about how to convert your figure files please see our guidelines: https://journals.plos.org/digitalhealth/s/figures

4. We notice that your supplementary materials are included in the manuscript file. Please remove them and upload them with the file type 'Supporting Information'. Please ensure that each Supporting Information file has a legend listed in the manuscript before or after the references list.

5. If the reviewer comments include a recommendation to cite specific previously published works, please review and evaluate these publications to determine whether they are relevant and should be cited. There is no requirement to cite these works unless the editor has indicated otherwise.

**Additional Editor Comments (if provided):** **Reviewers' Comments:**

**Comments to the Author**

1. Does this manuscript meet PLOS Digital Health’s publication criteria?

Reviewer #1: Yes

Reviewer #2: No

Reviewer #3: Yes

**********

2. Has the statistical analysis been performed appropriately and rigorously?

Reviewer #1: Yes

Reviewer #2: No

Reviewer #3: Yes

**********

3. Have the authors made all data underlying the findings in their manuscript fully available (please refer to the Data Availability Statement at the start of the manuscript PDF file)?

The PLOS Data policy

Reviewer #1: Yes

Reviewer #2: Yes

Reviewer #3: Yes

**********

4. Is the manuscript presented in an intelligible fashion and written in standard English?

Reviewer #1: Yes

Reviewer #2: No

Reviewer #3: Yes

**********

Reviewer #1: This study presented a highly relevant open-source intelligence system designed to track substandard and falsified medical products globally. The authors tackle a critical issue in global health security, and the development of this multilingual tool to make valuable contribution to the field. I recommend this manuscript for publication.

Reviewer #2: The study developed and evaluated a system to detect substandard and falsified (SF) medical products. However, the performance is not satisfactory, with the reported F1-score being only 52%. The methodology lacks clarity. The authors should provide additional information and conduct high-quality validation to ensure the reliability of the study. My specific comments are as follows:

In the Introduction, the authors state: "The extent of the problem is difficult to measure as most often the quality of a given medical product cannot be determined without specialised equipment and expertise in the relatively few reference laboratories globally." Given this, how can the Internet and electronic media be relied upon for accurate information about medical products? Please provide a clear rationale and supporting evidence for the study’s aims and practical implications.

Regarding data acquisition, how did the authors ensure data representativeness and reliability? On what basis was the search engine selected? How was fake news identified and verified? What measures were taken to assess the accuracy of fake news detection?

Please provide details of the technology/algorithm/method of the relevance classifier and related detection.

Why were different languages included in the study? Were any socio-cultural perspectives considered during the selection or analysis?

How many data analysts were involved in the labelling process? What was the level of agreement between analysts, and how was the labelling validated?

For Table 4: How were the reports validated to ensure they reliably described the medical products?

Reviewer #3: The manuscript is well written and presents an intriguing digital monitoring system. However, we could enhance the clarity, robustness, and future applicability of the proposed framework by improving a few aspects.

1. The automated relevance classifier shows a relatively low precision (Precision: 37%, Recall: 90%, F1 Score: 52%) compared to its recall. Although the high recall guarantees the capture of relevant reports, the lower precision could potentially lead to a significant number of false positives. The authors may consider discussing possible improvements, such as the use of more advanced natural language processing models (e.g., transformer-based approaches) to improve classification accuracy.

2. The performance of the georeferencing component appears relatively limited (Precision: 30%, Recall: 28%, F1 score: 29%). Since accurate location identification is critical for mapping incidents related to medicine quality, the authors may consider discussing potential improvements in geographic entity recognition methods.

3. The system relies heavily on publicly available news reports for identifying medicine quality incidents. Your study already quotes, "News data often remains unverified and should be interpreted cautiously." Since such reports may sometimes contain incomplete or unverified information, it would be beneficial for the authors to discuss possible validation mechanisms or cross-verification strategies.

4. The current system appears to depend significantly on specific news aggregation platforms. The authors may briefly discuss the potential limitations of such dependency and whether alternative data sources or APIs could be integrated in future versions of the system.

5. Although the system supports multiple languages, expanding the language coverage could further improve global monitoring capability. The authors may consider mentioning plans for integrating additional languages in future updates. As you have mentioned in your study on Indian fake news, India is the most populous country. Therefore, you might consider including Hindi and several other languages.

6. The manuscript indicates that duplicate reports across different languages may not always be automatically identified. Introducing cross-language similarity detection techniques could improve data consistency and reduce redundant entries.

7. The current workflow includes manual curation steps for validating and annotating reports. While this improves data quality, the authors may discuss possible semi-automated approaches that could improve scalability when processing large volumes of reports.

8. Future Real-Time Alert Capability: The system primarily functions as a monitoring and visualization platform. The usefulness of the system could be further enhanced by incorporating automated real-time alert mechanisms that notify relevant authorities when high-risk incidents are detected.

**********

what does this mean?). If published, this will include your full peer review and any attached files.

**Do you want your identity to be public for this peer review?**  If you choose “no”, your identity will remain anonymous but your review may still be made public.

For information about this choice, including consent withdrawal, please see our Privacy Policy

Reviewer #1: No

Reviewer #2: No

Reviewer #3: No

**********

**Figure resubmission:**

**Reproducibility:**

To enhance the reproducibility of your results, we recommend that authors of applicable studies deposit laboratory protocols in protocols.io, where a protocol can be assigned its own identifier (DOI) such that it can be cited independently in the future. Additionally, PLOS ONE offers an option to publish peer-reviewed clinical study protocols. Read more information on sharing protocols at https://plos.org/protocols?utm_medium=editorial-email&utm_source=authorletters&utm_campaign=protocols

Attachment Submitted filename: Minor Revision Comments.docx

# Author comment: Author response to Decision Letter 1

**DOI:** 10.1371/journal.pdig.0001648.r002

1 Jun 2026

Attachment Submitted filename: Minor Revision Comments - Responses - For Submission2.docx

# Peer review: Decision Letter 1

**DOI:** 10.1371/journal.pdig.0001648.r003

**Authors:** Jasmit Shah, Laura Sbaffi

22 Jul 2026

The Medicine Quality Monitoring Globe: A public health intelligence system for collecting and curating reports of substandard and falsified medical products from publicly available Internet news media

PDIG-D-26-00205R1

Dear Dr. Freifeld,

We are pleased to inform you that your manuscript 'The Medicine Quality Monitoring Globe: A public health intelligence system for collecting and curating reports of substandard and falsified medical products from publicly available Internet news media' has been provisionally accepted for publication in PLOS Digital Health.

Before your manuscript can be formally accepted you will need to complete some formatting changes, which you will receive in a follow-up email from a member of our team.

Please note that your manuscript will not be scheduled for publication until you have made the required changes, so a swift response is appreciated.

IMPORTANT: The editorial review process is now complete. PLOS will only permit corrections to spelling, formatting or significant scientific errors from this point onwards. Requests for major changes, or any which affect the scientific understanding of your work, will cause delays to the publication date of your manuscript.

If your institution or institutions have a press office, please notify them about your upcoming paper to help maximize its impact. If they'll be preparing press materials, please inform our press team as soon as possible -- no later than 48 hours after receiving the formal acceptance. Your manuscript will remain under strict press embargo until 2 pm Eastern Time on the date of publication. For more information, please contact digitalhealth@plos.org.

Thank you again for supporting Open Access publishing; we are looking forward to publishing your work in PLOS Digital Health.

Best regards,

Jasmit Shah, PhD

Academic Editor

PLOS Digital Health

***********************************************************

**Additional Editor Comments (if provided):**

The revised manuscript was evaluated by the original reviewers. Two reviewers consider that the authors have satisfactorily addressed all major concerns and now recommend publication. One reviewer recommended rejection, primarily due to concerns regarding the model's performance and suggestions for further methodological enhancement, including the potential use of large language models.

After carefully considering all reviewer reports, the revised manuscript, and the authors' responses, I find that the authors have adequately addressed the major issues raised during the previous rounds of review. The remaining concerns raised by the dissenting reviewer largely relate to suggestions for future improvements and possible extensions of the work rather than deficiencies that preclude publication. In particular, while improvements in predictive performance and exploration of alternative modelling approaches may strengthen future versions of the system, these are not essential requirements for demonstrating the current study's objectives within the scope of this manuscript.

On balance, I am satisfied that the manuscript now meets the journal's standards for methodological rigor and that the conclusions are appropriately supported by the data presented. Therefore, I am pleased to accept the manuscript for publication in PLOS Digital Health.

**Reviewer Comments (if any, and for reference):**

Reviewer's Responses to Questions

**Comments to the Author**

Reviewer #2: (No Response)

Reviewer #3: All comments have been addressed

**********

publication criteria?

Reviewer #2: No

Reviewer #3: Yes

**********

3. Has the statistical analysis been performed appropriately and rigorously?

Reviewer #2: No

Reviewer #3: Yes

**********

4. Have the authors made all data underlying the findings in their manuscript fully available (please refer to the Data Availability Statement at the start of the manuscript PDF file)?

The PLOS Data policy

Reviewer #2: (No Response)

Reviewer #3: Yes

**********

5. Is the manuscript presented in an intelligible fashion and written in standard English?

Reviewer #2: (No Response)

Reviewer #3: Yes

**********

Reviewer #2: The performance is unsatisfactory even during internal validation, and it is likely to decline further in external validation or real-world settings. It is important to enhance the performance and report the improvements in the manuscript. For instance, advanced analyses using large language models (LLMs) could be considered. However, the use of LLMs introduces additional ethical concerns that should be thoroughly addressed in the discussion.

Reviewer #3: (No Response)

**********

what does this mean?). If published, this will include your full peer review and any attached files.

**Do you want your identity to be public for this peer review?**  If you choose “no”, your identity will remain anonymous but your review may still be made public.

For information about this choice, including consent withdrawal, please see our Privacy Policy

Reviewer #2: No

Reviewer #3: No

**********
